# Reviews and Book Notices

**Published:** 1881-08

**Authors:** 


### Reviews and Book Notices.

ARTICLE XVI.—SEWER GAS AND ITS DANGERS. By George Preston Brown. Published by Jansen, McClurg & Co., Chicago.

This little book is well worth reading, if for no other reason that it may impress upon the mind of the reader the extent to which the drainage of this city is defective. The author pursued his investigations in conjunction with the Chicago Public Health Department. His personal investigations have been very extensive, and therefore, although not a physician, his observations and many of his conclusions are of value. There is no way of correcting the existing evil of defective drainage, except by spreading a general knowledge of the chief faults in the present systems, and the best modes of correcting them. This is the object of the little volume under review. Fully one-half of the contents is taken up with descriptions of house drains which were allowing the escape of gas, that was plainly one, if not the chief, cause of the diseases which attacked the inhabitants. The faults pointed out, every reader will see, exist in a very large number of the Chicago houses. The conclusions which Mr. Brown arrives at in regard to the requirements of a perfect system it may be well to copy here.

1. There must be an unobstructed and positive ventilation of all the pipes and drains within and beneath the house.

2. The ordinary earthenware drains must be discarded, since they cannot be secured against breaking and defective joints.

3. Perfect joints and not mere connections must be made between sections of drains and pipes.

4. The waste-pipes and drains must be one continuous system, and made of material that will not break, leak, corrode nor obstruct the free passage of the minutest particles which enter them.

5. Traps must be used that will not permit the escape of gas by defect in construction or use; or, better still, they should be entirely discarded.

All should remember that the best disinfectant and the best insurance against disease is pure, fresh air.

---

RACHITIS AND CALCIUM PHOSPHATE. By Dr. Des Vallères.

The introduction of phosphate of lime in the materia medica is not recent. On perusing old pharmacopœia, we repeatedly meet formulæ containing burnt hartshorn, lobster's eyes, oyster shells, *album græcum*, etc., which consist almost entirely of calcium phosphate. Physiological experiments lately performed with that substance, by Drs. Chossat, Lassaigne, Cazalis, Guérin, Piorry, Gosselin, and many others, gave results which justified the reputation it held in the opinion of our predecessors.

The properties of calcium phosphate, brought to light by the learned investigators above referred to, may be thus summed up: *It has an immediate effect on the consolidation and the reparation of bones; at the same time, and above all, it acts as an excitant of assimilation*—an important property, for what we digest and absorb, not what is ingested, nourishes the body. An idea, which could hardly fail to suggest itself, and it is a happy one, consists in associating peptine with calcium phosphate, after this has been rendered soluble and been changed into chlorhydrophosphate. I have been very much satisfied with it personally, and I have seen very good results follow its use, in puny children, lacking in energy, suffering from obstinate eye and ear troubles, from adenitis, very much predisposed to colds, etc. In a word, in all the sufferers from a scrofulous diathesis.

On the same principle, there is no doubt that phosphated peptone should be prescribed for pregnant women, who must furnish the fœtus with the necessary phosphates, beside its other elements. It is a well-known fact that, during gestation, the bones of the mother lose in their firmness; something, not so well known, is the report of Dr. Pégot-Ogier on that point. Here are the conclusions at which he arrived:

It results from the use of calcium phosphate that:

"1. In pregnant women, most of the common ailments pertaining to their condition disappear, and the number of still-births is diminished.

"2. The mother's milk, often deficient in calcario-phosphatic elements, attains its maximum of richness which nature has fixed for the child.

"3. During infancy, youth, and until adult age, the development of the body progresses regularly; lymphatic nor rachitic diseases need be feared.

"4. The death rate, which, in Paris, is one to three (during the first year), has been brought down to one in five, which is the rate observed in the most salubrious places of the country."

Poor and sickly nursing children are common indeed; this most often is owing to one of two causes; either the mother's milk is too poor, or it is not well assimilated, the immediate result being an impaired digestion, which is often manifested by chronic diarrhœas, with wasting, and a slow alteration of the economy, which may lead to rachitis. In that case, nothing is so rational as the administration of phosphated peptones to the nurse, and this will bring the milk to its normal standard, and furnish the child with the stimulant of nutrition, besides the elements necessary for the formation of the bones and teeth.

According to Liebeg and Humboldt, crude calcareous mixtures are administered to children to facilitate their *growth* and their *dentition*, in Germany.

Indeed, I might insist on the benefits which we can expect from that preparation in all diseases of the bones: caries, necroses, osteo-malacia, Pott's disease, etc.; but I could not omit that to these diseases in particular does Trousseau's recommendation apply: "Choose especially the time when the constituents of the child's body are undergoing rapid changes, to renovate its constitution."—*L' Union Médical*, March 1.

